# Honokiol acts as an AMPK complex agonist therapeutic in non-alcoholic fatty liver disease and metabolic syndrome

**DOI:** 10.1186/s13020-023-00729-5

**Published:** 2023-03-17

**Authors:** Ruifeng Tian, Jinjie Yang, Xiaoming Wang, Shuaiyang Liu, Ruixiang Dong, Zhenya Wang, Zifeng Yang, Yingping Zhang, Zhiwei Cai, Hailong Yang, Yufeng Hu, Zhi-Gang She, Hongliang Li, Junjie Zhou, Xiao-Jing Zhang

**Affiliations:** 1 Department of Cardiology,Renmin Hospital; School of Basic Medical Science, Wuhan University, Wuhan, 430060 China; 2 Institute of Model Animal of Wuhan University, Wuhan, 430071 China; 3 School of Pharmacy, Bengbu Medical College, Bengbu, 233030 China; 4 Gannan Innovation and Translational Medicine Research Institute, Gannan Medical University, Ganzhou, 341000 China; 5 Key Laboratory of Prevention and Treatment of Cardiovascular and Cerebrovascular Diseases, Ministry of Education, Gannan Medical University, Ganzhou, 341000 China; 6 Medical Science Research Center, Zhongnan Hospital of Wuhan University, Wuhan, 430071 China

**Keywords:** Honokiol, AMPKγ1, Non-alcoholic fatty liver disease, Obesity

## Abstract

**Background:**

Non-alcoholic fatty liver (NAFLD) and its related metabolic syndrome have become major threats to human health, but there is still a need for effective and safe drugs to treat these conditions. Here we aimed to identify potential drug candidates for NAFLD and the underlying molecular mechanisms.

**Methods:**

A drug repositioning strategy was used to screen an FDA-approved drug library with approximately 3000 compounds in an in vitro hepatocyte model of lipid accumulation, with honokiol identified as an effective anti-NAFLD candidate. We systematically examined the therapeutic effect of honokiol in NAFLD and metabolic syndrome in multiple in vitro and in vivo models. Transcriptomic examination and biotin-streptavidin binding assays were used to explore the underlying molecular mechanisms, confirmed by rescue experiments.

**Results:**

Honokiol significantly inhibited metabolic syndrome and NAFLD progression as evidenced by improved hepatic steatosis, liver fibrosis, adipose inflammation, and insulin resistance. Mechanistically, the beneficial effects of honokiol were largely through AMPK activation. Rather than acting on the classical upstream regulators of AMPK, honokiol directly bound to the AMPKγ1 subunit to robustly activate AMPK signaling. Mutation of honokiol-binding sites of AMPKγ1 largely abolished the protective capacity of honokiol against NAFLD.

**Conclusion:**

These findings clearly demonstrate the beneficial effects of honokiol in multiple models and reveal a previously unappreciated signaling mechanism of honokiol in NAFLD and metabolic syndrome. This study also provides new insights into metabolic disease treatment by targeting AMPKγ1 subunit-mediated signaling activation.

**Supplementary Information:**

The online version contains supplementary material available at 10.1186/s13020-023-00729-5.

## Background

Non-alcoholic fatty liver disease (NAFLD) is a collection of liver disorders ranging from simple steatosis (fatty liver) to non-alcoholic steatohepatitis (NASH) with necroinflammation and progressive fibrosis [1]. NAFLD is now an established and increasing cause of mortality and morbidity from liver disease, with in silico modeling predicting a significant increase in disease (and consequently economic) burden over the next decade, especially as the prevalence of obesity grows worldwide [2–5]. NAFLD can also have other, non-liver-related negative impacts, especially on cardiovascular health [6, 7]. However, there are still no clinically-approved drugs for NASH and discrepancies in the results from animal models and safety concerns limit the translation of laboratory findings to the clinic. Therefore, there is an urgent need for drug development in this area.

AMP-activated protein kinase (AMPK) is a sensor of cellular energy status, nutrient availability, and cellular injury implicated in the pathogenesis of cardiovascular disease, chronic metabolic disease, and cancer [8]. The AMPK complex is formed of mandatory heterotrimers consisting of the catalytic α subunit, the scaffold β subunit, and the regulatory γ subunit. Different subunit isoforms have specific spatiotemporal tissue and cellular expression patterns, so the pathophysiological regulation of AMPK is highly complex [9]. Activating AMPK has been shown to protect against NAFLD and metabolic syndrome, but chronic activation might also have serious adverse sequelae in the form of cardiac hypertrophy and cancer [10]. These effects may in part be due to AMPK activators mainly targeting the AMPK α and β subunits that possess non-substitutable capacity in regulating pathophysiological behaviors. However, AMPKγ is well conserved in eukaryotes and archaea [11], so it may be a good translational target. Encouragingly, a recent study reported that liver gain-of-function mutations in the AMPKγ1 subunit protected against hepatic steatosis [12]. However, there have been relatively few studies of AMPKγ1-targeting drugs, and their development and testing in NAFLD and metabolic syndrome requires further study.

FDA-approved drug libraries include drugs that have been shown to be clinically effective and drugs with known pharmacological activity included in pharmacopoeias [13]. Importantly, these drugs have been shown to be safe in the clinical setting. In our pharmacological screening, honokiol, an active ingredient found in the traditional Chinese herb magnolia [14], was one of the most effective drug candidates for NASH therapy. Honokiol belongs to a class of neolignan biphenols, and it has been shown to have anti-inflammatory, anti-infection, anti-oxidative, and anti-tumor effects [15–18]. Indeed, natural polyphenol is also reported to show antioxidative effect in liver [19]. Honokiol is also relatively non-toxic in experimental contexts, and several food safety authorities have evaluated honokiol as safe [20]. Previous reports have suggested that honokiol is beneficial in hepatocyte lipotoxicity and macrophage polarization [21–24], but it remains unclear whether honokiol could be a drug candidate for treating the spectrum of NASH and related metabolic syndrome diseases. Moreover, the molecular mechanisms underpinning honokiol's protective effect are still not fully understood.

The aim of this study was to screen for effective drugs targeting NASH and establish the underlying molecular mechanisms. To do so, we combined in vitro and in vivo modeling to systematically examine the protective effects of honokiol in NASH and the accompanying metabolic features. First, we screened an FDA-approved drug library in an in vitro hepatocyte model of lipid accumulation, in which honokiol exerted significant efficacy. To further evaluate the underlying therapeutic mechanisms, we administered honokiol to murine models of NAFLD induced by a high-fat diet (HFD) and NASH induced by choline-deficient, L-amino acid-defined (CDA)HFD or methionine-choline deficient (MCD) diets. Transcriptomic analysis revealed a role for AMPK activation in honokiol's mechanism of action, which was further validated using pharmacological and genetic approaches. While classical regulators of AMPK activation did not appear to be implicated in its honokiol-mediated regulation, docking analysis predicted that honokiol could directly bind to AMPKγ1, which was subsequently confirmed experimentally. We therefore show that honokiol exerts its protective effects through AMPK activation via a new activating mechanism. These findings represent a significant step towards the discovery of a new class of drugs that target AMPK to manage NAFLD and NASH.

## Methods and materials

### Cell lines and primary hepatocytes

Cell lines of L02 and HEK293T were obtained from the Chinese Academy of Sciences in Shanghai, China. L02 and HEK293T cells were cultivated in a Dulbecco's modified Eagle medium (DMEM) enriched with 10% FBS and 1% penicillin/streptomycin.

Two-step collagenase perfusion was used to acquire primary hepatocytes from 6- to 8-week male C57BL/6 J mice. Briefly, mice were sedated with 3% pentobarbital sodium (90 mg/kg, #P3761, Sigma-Aldrich, St. Louis, MO). The anesthetized mice were sequentially perfused via the portal vein with Liver Perfusion Medium (#17701038, Thermo Fisher Scientific, Waltham, MA) and Liver Digestion Medium (#17701034, Thermo Fisher Scientific). The livers were then removed, chopped into small pieces, and passed through a 100 µm steel mesh. After two centrifugations at 50 × g for a duration of one-minute, primary mouse hepatocytes were isolated from a mixture of liver cells.

To recreate lipid accumulation in vitro, L02 cells or primary hepatocytes were cultured with a medium composed of 500 μM palmitic acid (PA) and 1 mM oleic acid (OA) and treated with honokiol (10 μM) or DMSO. 500 μM PA was used to construct a model of hepatocytes inflammation. To serve as a control, a 0.5% BSA was employed. 10 μM compound C (CC) was used to inhibit AMPK phosphorylation.

*PRKAA1/PRKAA2*-deficient cell lines were generated using the CRISPR/Cas9 system as described in our previous work [25]. AMPKγ1 knockdown cell lines were generated by cloning a short hairpin sequence (GTCTTGTCCTCTAGGCATGCT) targeting human *PRKAG1* into the pLKO.1 plasmid (#10878, Addgene, Watertown, MA). The hairpin sequence targeting *PRKAG1* was designed using an online tool (http://rnaidesigner.thermofisher.com/rnaiexpress/design.do). The short hairpin RNA-expressing plasmid was combined with the packaging plasmids pMD2.G (#12259, Addgene) and psPAX2 (#12260, Addgene) at a ratio of 2:1:1 and co-transfected into HEK293T cells. Following transfection, the supernatants were harvested after 48 h and filtered through a 0.45 µm filter. L02 hepatocytes were then infected with the collected supernatants with the help of polybrene (2 mg/mL). To screen positive candidates, infected cells were killed with puromycin (1 μM).

### High-content screening from FDA-approved drug library

To identify potential effective drugs from the FDA-approved drug library (#L1300, Selleckchem, USA), we employed the human hepatocyte cell line L02. The FDA-approved drugs library was procured from Selleck. 10,000 hepatocytes were seeded per well in a 96-well plate. The day following plating, we exposed the cells to 0.5 mM/1 M PA/OA for 18 h while simultaneously administering the drugs. The drugs were administered at a concentration of 20 μM. Bodipy staining was employed to assess lipid accumulation, and the fluorescence intensity was then quantified using a high-content machine.

### Animal experiments

The male C57BL/6 J mice were provided free access to food and water in a temperature-controlled environment (23 ± 2 °C). C57BL/6 J mice were fed a high-fat diet (HFD) (#MD12032, Medicience, Jiangsu, China) starting at 8 weeks age to establish a NAFLD model. After HFD feeding for 12 weeks, mice were divided evenly into two groups, one of which received vehicle (1% carboxymethylcellulose sodium (CMC-NA), # 419273, Sigma-Aldrich) or 100 mg/kg honokiol (#BD8971-25 g, Bidepharm, Shanghai, China. The chemical purity was 98%) dissolved in 1% b-CMC-NA by gavage every day. At 24 weeks after HFD feeding, mice were sacrificed and blood, liver, and white adipose tissues (WAT) samples were collected for further study.

To create a NASH model, C57BL/6 J mice were given a choline-deficient, L-amino acid-defined (CDA)HFD (A06071302, Research Diets, Inc., New Brunswick, NJ) or methionine-choline deficient (MCD) diet (TP3005G, Trophic Animal Feed High-Tech Co, Nantong, China) beginning at the age of 8 weeks. After feeding CDAHFD or MCD for 1 week, mice were separated evenly into two groups, which respectively received 1% CMC-NA or 100 mg/kg honokiol dissolved in 1% CMC-NA by gavage every day. After feeding 4 weeks for indicated diets, animals were sacrificed and their blood and liver samples were collected for further analysis.

To demonstrate the in vivo requirement for AMPK activation-mediated honokiol protection against NASH, C57BL/6 J mice were fed a CDAHFD for 1 week, and then assigned randomly to two groups, and treated with PBS or AMPK inhibitor compound C (CC, 10 mg/kg/every two days) in combination with 1% CMC-NA or honokiol (100 mg/kg/every day) for another 3 weeks. After being fed for four weeks, mice were sacrificed and their blood and liver samples were taken for further analysis.

### Glucose and insulin tolerance tests

Glucose tolerance tests (GTT) were performed in mice after 22 weeks of HFD feeding or after 3 weeks of the CDAHFD diet. Following an 18 h fast, mice were intraperitoneally (i.p.) injected with 1 g/kg glucose. Subsequently, blood glucose levels were monitored at 0, 15-, 30-, 60-, and 120-min post-injection.

Following 23 weeks of high-fat diet feeding, insulin tolerance tests (ITT) were performed on the mice. 0.75 IU/kg insulin was injected intraperitoneally after a six-hour fast and their blood glucose levels were measured at intervals of 0, 15-, 30-, 60-, and 120-min post-injection.

### Serum biochemical analysis

Serum alanine transaminase (ALT), aspartate transaminase (AST), total cholesterol (TC), and triglycerides (TG) were detected to evaluate liver function and serum concentrations of lipids using an automatic biochemical analyzer (HITACHI 3110, Tokyo, Japan).

### Western blotting

Proteins were extracted from cells or mouse liver tissues using RIPA lysis buffer, which contained 50 mM Tris–HCl pH 8.0, 150 mM NaCl, 1 mM EDTA, 1% NP-40, 0.5% sodium deoxycholate and 0.1% SDS. The extraction process included the use of complete protease inhibitor cocktail tablets (#04693132001, Roche, Basel, Switzerland) and phosphatase inhibitor (#4906837001, Roche). The concentration of samples was then determined with a BCA Protein Assay Kit (#23225, Thermo Fisher Scientific). Protein samples were fractionated via sodium dodecyl sulfate polyacrylamide gel electrophoresis (SDS-PAGE) and then transferred to 0.45 µm PVDF membranes. Following the blocking the membranes with 5% skimmed milk, primary antibodies were incubated overnight at 4℃, followed by 1 h incubation with secondary horseradish peroxidase (HRP)-conjugated antibodies at room temperature. Finally, the protein expression signals were detected in a ChemiDoc MP Imaging System (Bio-Rad, Hercules, CA). β-actin was used as an internal control for loading.

### Antibodies

Primary antibodies targeting ACC (#3676), p-ACC (#3661), AMPKα (#5832), p-AMPKα (#50081), mTOR (#2983), p-mTOR (#2971), CaMKK2 (#16,810), p-CaMKK2 (#12818), TAK1 (#4505), p-TAK1 (#4508), LKB1 (#3050), and p-LKB1 (#3055) were procured from Cell Signaling Technology (Danvers, MA). Primary antibodies targeting PP2C (#ab211660) were procured from Abcam (Cambridge, UK). Antibodies targeting actin were obtained from ABclonal (AC026, 1:3000; Wuhan, China). Antibodies targeting Flag (M185-3L) were obtained from MBL (Nagoya, Japan). Unless otherwise specified, the dilution of all primary antibodies was 1:1000. The secondary antibodies peroxidase AffiniPure goat anti-rabbit IgG (H + L) (111–035-003) and goat anti-mouse IgG (H + L) (115–035-003) were obtained from the Jackson Laboratory (Bar Harbor, ME). A 1:5000 dilution was used for secondary antibodies.

### Lipid droplet staining and detection

Cellular lipid droplets were measured by BODIPY (D3922, Thermo Fisher Scientific) staining. L02 or primary hepatocytes were fixed with 4% paraformaldehyde for 20 min at room temperature after challenge with PA/OA for 12 h. Following a PBS wash, cells were subjected to BODIPY staining at room temperature for 15 min. Cellular nuclei were stained with 4',6-diamidino-2-phenylindole (DAPI) (S36939, Invitrogen, Waltham, MA). A confocal laser scanning microscope (TCS SP8X, Leica, Wetzlar, Germany) was used to acquire images.

### Cell viability determination

Primary hepatocytes were seeded in 96-well plates at 5000 cells per well and incubated with 10 μM of honokiol for 24 h. Next, 10 μL of Cell Counting Kit-8 reagent (Beyotime, China) was added to each well and incubated for an additional 4 h at 37 ℃. Absorbance at 450 nm was measured for each well to calculate cell viability.

### Detection of ATP, ADP, and AMP

After honokiol (100 mg/kg) administration 4 h, mice were anesthetized and their livers were quickly frozen and clamped to quantify AMP, ADP, and ATP levels. Utilizing 50 mg liver tissue from each mouse, ATP, ADP, and AMP were extracted and the detection parameters were previously specified [26].

### TG or TC detection

To determine hepatic lipid contents, cellular lipid was extracted from 50 mg of liver tissue using the Folch method as previously described [27]. The liver was tested for triglycerides (TG), total cholesterol (TC), and non-esterified fatty acids (NEFA) using Wako kits (Tokyo, Japan) as per the manufacturer’s instructions (#290–63701 for TG, #294–65801 for TC, #294–63601 for NEFA).

### Cellular respiration evaluation

The effect of honokiol on cellular respiration was assessed in primary mouse hepatocytes. Single cell suspensions of 0.1 M primary hepatocytes were prepared in DMEM with honokiol (10 μM) or DMSO. The oxygen consumption rate was documented after the successive administration of oligomycin (2.5 mM), FCCP (0.5 mM), rotenone (0.5 mM) and antimycin A (2.5 mM).

### Histopathological analysis

For histopathological analysis, HE staining (hematoxylin, G1004, Servicebio, Wuhan, China; eosin, BA-4024, Baso, Zhuhai, China) was examined on liver, heart or adipose tissues (AT). Liver lipid droplets were observed by oil red O (O0625, Sigma-Aldrich) staining using frozen liver tissues embedded in Tissue-Tek OCT Compound (4583, Sakura, Torrance, CA). Liver fibrosis was observed with picro-sirius red (26357–02, Hedebiotechnology, Beijing, China) staining. Histopathological images were acquired with a light microscope (ECLIPSE 80i, Nikon). The NAFLD activity scoring (NAS) system was used to quantify NAFLD in HE-stained liver sections [28]. Other histological images were quantified using Image-J.

### Immunohistochemistry

Immunohistochemistry staining of liver CD11b (BM3925, 1:12000 dilution; Boster; Wuhan, China) and WAT F4/80 (GB11027, 1:1600 dilution, Servicebio, Wuhan, China) were performed on paraffin embedded sections. To retrieve antigens, samples were boiled in a pressure cooker for 20 min in pH 9.0 EDTA buffer. After cooling, samples were placed in 3% H_2_O_2_ for 20 min to quench endogenous peroxide activity. Following a wash with PBS, slides were blocked with 10% BSA for 1 h at 37 ℃. Sections were incubated with the indicated primary antibodies overnight at 4℃. The next day, the sections were successively washed with PBS buffer for 5 min, followed by incubation with enhanced enzyme-labeled goat anti-rabbit IgG (Beijing ZSGB Biotech) at 37 degrees Celsius for one hour. Immunohistochemical staining was performed using the 3,30-diaminobenzidine (DAB) substrate kit (Beijing ZSGB Biotech) and counterstained with hematoxylin. The images were then taken with a light microscope. Histological images were quantified using Image-J.

### Synthesis of biotin-linked honokiol


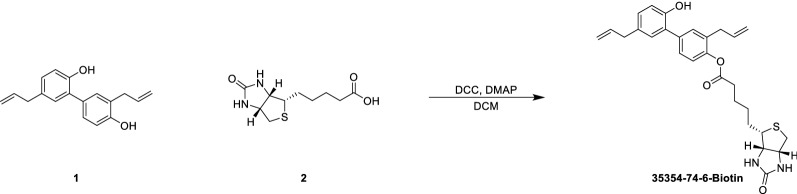


A mixture of honokiol (270 mg, 1 eq) in dichloromethane (DCM) (5 mL) was added to biotin (378 mg, 1.5 eq), 4-dimethylaminopyridine (DMAP) (126 mg, 1 eq), and N, N’-dicyclohexylcarbodiimide (DCC) (522.9 mg, 2.5 eq). The reaction mixture was stirred at 25 °C for 12 h. LCMS showed a new peak of the desired mass. The mixture was poured into water (20 mL), extracted with EA (15 mL × 3), washed with saline (20 mL × 2), and concentrated under high vacuum to produce a residue. The residue was purified with a flash C18 column (20–63% ACN, 0.1% TFA) and lyophilization to produce biotin-linked honokiol (80 mg, 95% purity) as a white solid.

### Biotin-avidin binding assay

Plasmids expressing AMPKγ1, AMPKγ2, and the AMPKγ1-3A mutant were cloned into phage vectors. Cloned sequences were confirmed by Sanger sequencing. 293 T cells were seeded in 10 cm cell culture dishes. At 70% confluence, 293 T cells were transfected with the indicated plasmids (12 μg). After 24 h, biotin-linked honokiol (40 μM) or biotin was added and incubated for another 4 h. The dish was washed with precooled PBS, and 1 ml immunoprecipitation buffer containing protease inhibitor cocktail tablets and phosphatase inhibitor tablets were added to lysis. To remove cellular debris, the lysates were centrifuged at 12,000 × *g* for 15 min at 4℃. Supernatants were incubated with Streptavidin Agarose Resins (#20353, Thermo Fisher Scientific) at 4 °C for 4 h followed by washing 5 times in cold immunoprecipitation wash buffer. The protein complex pull-down was degenerated in SDS loading buffer and subjected to western blot analysis using the indicated primary and corresponding secondary antibodies as described above.

### RNA-sequencing

The quality of extracted RNA samples was evaluated using the RNA 6000 Nano kit (#5067–1511, Agilent, Santa Clara, CA) after extraction with TRIzol reagent (#T9424, Sigma-Aldrich). In order to prepare the libraries, we used the MGIEasy RNA Library Prep Kit (#1000006384, MGI Tech Co., Ltd, Shenzhen, China).

For data analysis, sequences from cleaned reads were aligned to the Ensembl GRCm38 mouse genome with HISAT2, and SAMtools was used to sort and convert the mapped reads to BAM format. RAW counts and reads per kilobase per million (RPKM) values were calculated for each gene with StringTie. Normalized counts and differential expression between conditions were calculated with DESeq2 (v1.32.0). Differentially expressed genes (DEGs) were identified as those with |log2 (fold change) |≥ log2(1.5) and an adjusted *P*-value < 0.05. GSVA was carried out using the GSVA R package (v1.40.1) to assess pathway activity variation under different conditions. Gene sets with *P-*values < 0.05 were considered statistically significant.

### Statistical analysis

All data were analyzed using SPSS v26 and are expressed as the means ± SEM. For parametric data between two groups, a Student’s *t*-test was used to analyze differences. For parametric data for multiple comparisons, a one-way ANOVA was performed. Bonferroni's post hoc test was employed to analyze data that demonstrated significant results, while Tamhane's T2 (M) post hoc test was used for heteroscedastic data. For datasets with a skewed distribution, the Mann–Whitney U test and Kruskal–Wallis test were utilized for two and multiple group comparisons, respectively. *P*-values < 0.05 were considered significant.

## Results

### Screening of an FDA-approved drug library reveals honokiol is a candidate inhibitor of NAFLD

**Fig. 1:**
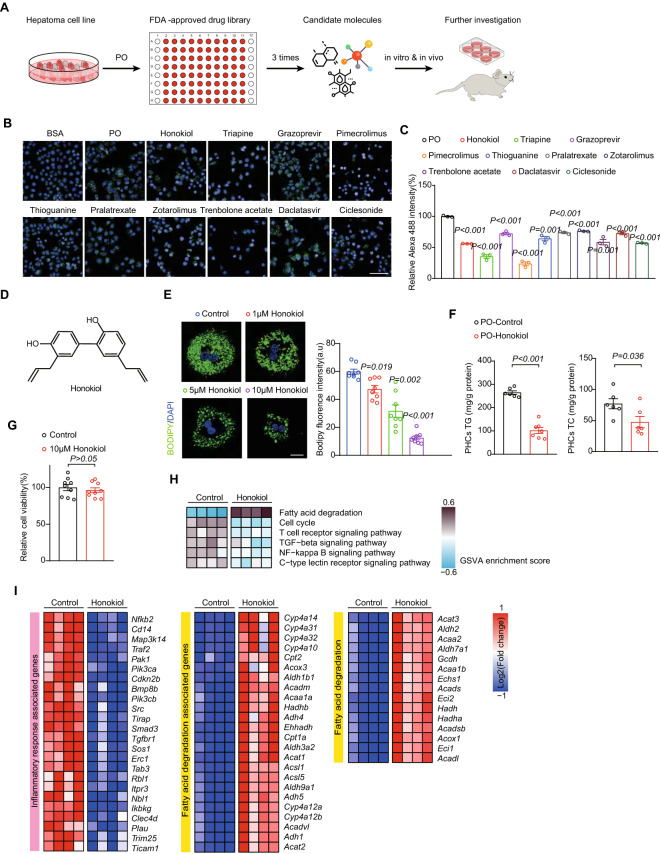
Screening candidate drugs in an FDA-approved library for lipid-lowering effects in hepatocytes and evaluating the therapeutic effects of honokiol in vitro. **A** Schematic of the screening strategy used with the FDA-approved drug library. **B** and **C** Representative image (**B**) and relative Alexa-488 intensity (**C**) of the top 10 candidates. n = 3 replicates. Student’s *t*-test was applied for statistical analysis. Scale bar 25 μm. **D** The molecular structure of honokiol. **E** Representative images (left) and quantification of fluorescence (right) generated by BODIPY staining of primary hepatocyte lipid droplets after treatment with different concentrations of honokiol. n = 3 replicates. Student’s *t*-test was applied for statistical analysis. Scale bar 10 μm. **F** Triglyceride (TG) and total cholesterol (TC) in primary hepatocytes challenged with PO stimulation for 12 h. n = 6 mice. Student’s *t*-test was applied for statistical analysis. **G** Relative cell viability of primary hepatocytes after treatment with 10μM honokiol for 24 h. n = 3 replicates. Student’s *t*-test was applied for statistical analysis. **H** GSVA enrichment of differentially regulated pathways involved in cell growth, lipid degradation, and immune responses in primary hepatocytes after treatment with PO for 12 h. **K** Heatmaps of gene expression associated with inflammation, and lipid degradation in primary hepatocytes after treatment with PO for 12 h

To effectively screen for drugs to prevent or treat NAFLD, we screened an FDA-approved drug library using L02 human hepatocytes in vitro*.* The therapeutic effect of drug candidate was further evaluated in multiple mice models (Fig. 1A). The cultured cells were exposed to palmitic/oil acid (PO) for 18 h to induce lipid accumulation, and compounds in the library were added at the same time with PO challenge. A high-content instrument was used to evaluate the lipid-lowering effect of the FDA library, and the top 10 candidates were further compared in independent experiments (Fig. 1B, C). Finally, honokiol was identified as an attractive potential candidate through a series of screenings of ~ 3000 drugs (Fig. 1D). Using a primary mouse hepatocyte model, we further confirmed the lipid-lowering and anti-inflammatory effects of honokiol with well-tolerable safety. BODIPY staining and TG and TC colorimetric assays showed that honokiol significantly decreased lipid droplet accumulation in the presence of PO stimulation (Fig. 1E, F). Cell viability assays indicated that current working concentrations had little to no effect on cells (Fig. 1G). Furthermore, transcriptomic analysis of primary hepatocytes showed robust inhibition of pathways and genes associated with inflammatory responses, and upregulated pathways and genes related with and fatty acid degradation ( Fig. 1H, I).

### Honokiol ameliorates high-fat diet (HFD)-induced NAFLD

**Fig. 2:**
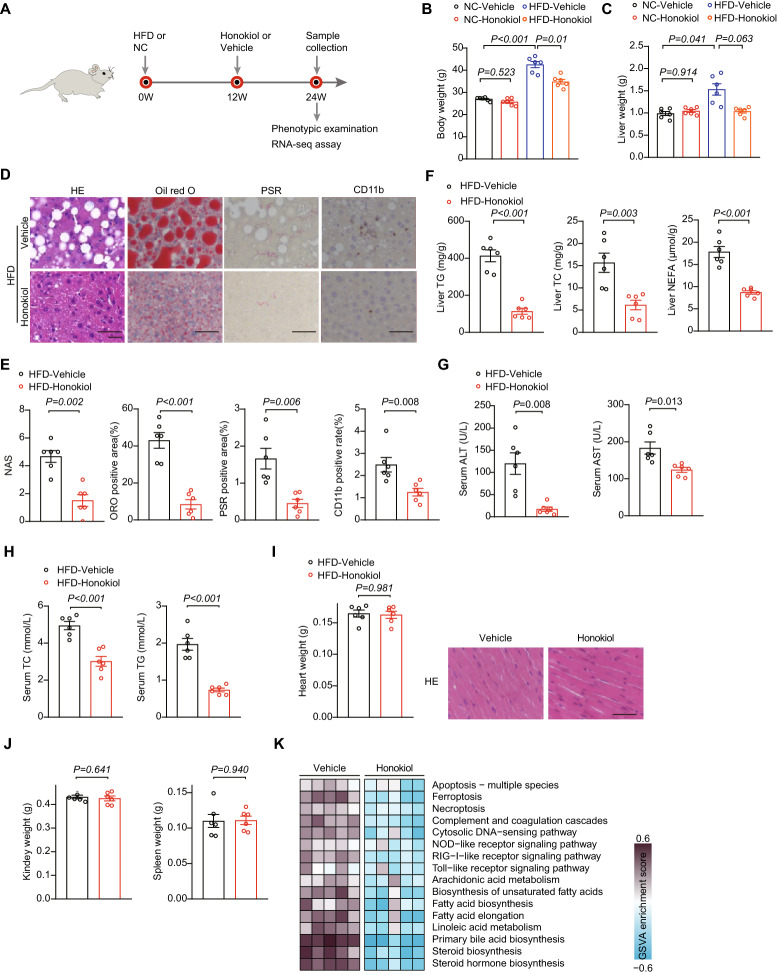

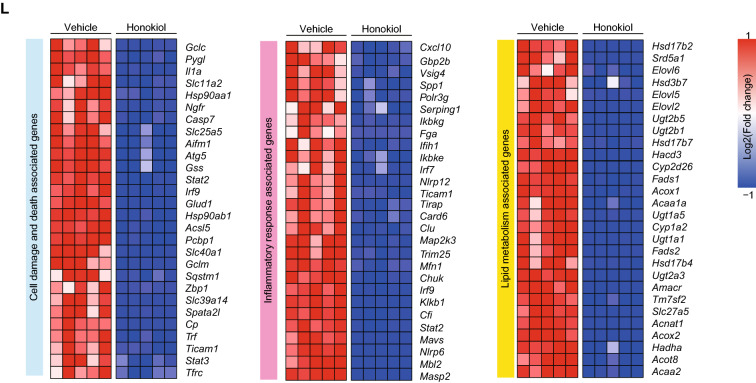
Honokiol ameliorates high fat diet (HFD)-induced non-alcoholic fatty liver disease. **A** Schematic showing HFD-induced NAFLD and evaluation of therapeutic effects of honokiol in vivo (100 mg/kg). **B** and **C** Body (**B**) and liver weight (**C**) of NC- or HFD-fed mice treated with honokiol or vehicle after 12 weeks of their respective diets. n = 6 mice per group. One-way ANOVA was used for statistical analysis. **D** Representative images of indicated mouse liver sections stained with hematoxylin and eosin (HE), oil red O (ORO), picrosirius red (PSR), and immunohistochemistry (IHC) of CD11b-positive cells. n = 6 mice per group. Scale bar 50 μm. **E** Results of NAS (HE) and quantitative analysis of ORO, PSR, and CD11b shown in (**D**). n = 6 mice per group. Mann–Whitney U test was used for NAS, while Student’s *t*-test was applied to ORO, PSR, and CD11b data. **F** TG, TC, and non-esterified fatty acids (NEFA) in the livers of HFD-fed mice treated with honokiol or vehicle after 12 weeks of their respective diets. n = 6 mice per group. Student’s *t*-test was applied for statistical analysis. **G** Serum ALT and AST activity in HFD-fed mice treated with honokiol or vehicle after 12 weeks of their respective diets. n = 6 mice per group. Student’s *t*-test was applied for statistical analysis. **H** Serum TC and TG concentrations in HFD-fed mice treated with honokiol or vehicle after 12 weeks of their respective diets. n = 6 mice per group. Student’s *t*-test was applied for statistical analysis. **I** Heart weights and heart histological staining of HFD-fed mice treated with honokiol or vehicle after 12 weeks of their respective diets. n = 6 mice per group. Student’s *t*-test was applied for statistical analysis. Scale bar 50 μm. **J** Kidney and spleen weights of HFD-fed mice treated with honokiol or vehicle after 12 weeks of their respective diets. n = 6 mice per group. Student’s *t*-test was applied for statistical analysis. **K** GSVA enrichment analysis related to inflammation, lipid metabolism, and fibrosis downregulated by honokiol treatment. n = 5 mice per group. **L** Heatmap of gene expression profiles involved in cell damage and death, inflammation, and lipid metabolism. n = 5 mice per group

To explore the potential clinical relevance of these findings, we evaluated the therapeutic effect of honokiol in HFD-fed mice. Mice were subjected to normal chow (NC) or a HFD for 12 weeks to initiate the NAFLD phenotype and were then treated with vehicle (carboxymethylcellulose, 1% CMC) or honokiol (100 mg/kg/ every day) for an additional 12 weeks (Fig. 2A). The administration of honokiol significantly attenuated the increase in body weight, and there was a trend towards decreasing the liver weight gain induced by HFD (Fig. 2B, C). Histopathological analysis of liver tissue stained for lipids showed that honokiol treatment significantly decreased the size and contents of hepatic lipid droplets compared with vehicle treatment. In addition, honokiol significantly reduced the severity of fibrosis and inflammatory cell infiltration (Fig. 2D, E). The results of direct detection of liver TG, TC, and NEFA were consistent with the histological findings (Fig. 2F). Moreover, the liver injury markers (ALT and AST) and serum TC and TG were significantly reduced in the honokiol-treated group (Fig. 2G, H). There was no evidence of adverse effects on heart, kidney, or spleen function ( Fig. 2I, J). Global transcriptome analysis of HFD mouse livers showed significant improvements in cell damage, inflammation, and lipid accumulation pathways in honokiol-treated mice (Fig. 2K, L). We conducted a further investigation into the lipid metabolism pathway impacted by honokiol treatment and discovered that honokiol usage significantly curbed several lipid metabolism pathways (Additional file 1: Fig. S1A).

### Honokiol ameliorates HFD-induced obesity and insulin resistance

**Fig. 3:**
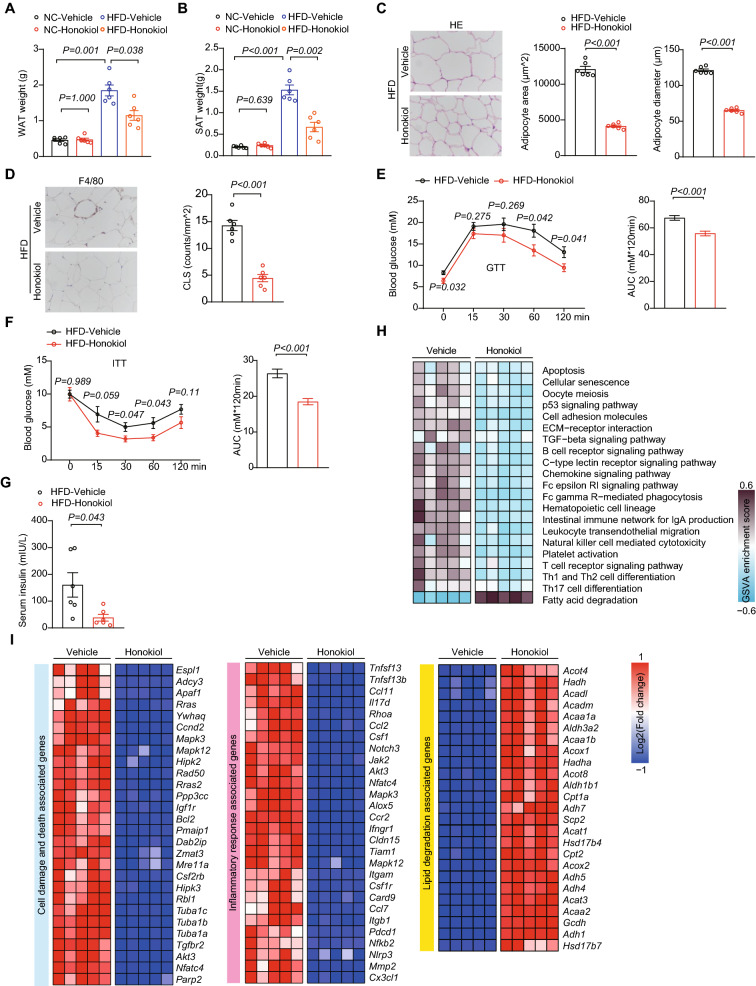
Honokiol ameliorates high fat diet (HFD)-induced obesity and insulin resistance.** A** and **B** Weights of white adipose tissue (WAT) (**A**) and subcutaneous adipose tissue (SAT) (**B**) from NC- or HFD-fed mice treated with honokiol or vehicle after 12 weeks of their respective diets. n = 6 mice per group. One-way ANOVA was used for statistical analysis. **C** Representative images and quantification of HE staining of WAT from HFD-fed mice treated with honokiol or vehicle after 12 weeks of their respective diets. n = 6 mice per group. Student’s *t*-test was applied for statistical analysis. Scale bar 50 μm. **D** Representative images and quantification of IHC staining of F4/80-positive cells in WAT sections. CLS, crown-like structure. n = 6 mice per group. Student’s *t*-test was applied for statistical analysis. Scale bar 50 μm. **E** Blood glucose concentrations during glucose tolerance testing (GTT) of HFD-fed mice treated with honokiol or vehicle at 22 weeks. n = 6 mice per group at each time point. Student’s *t*-test was applied for statistical analysis. Area under the GTT curve shown right. **F** Blood glucose concentrations during insulin tolerance testing (ITT) of HFD-fed mice treated with honokiol or vehicle at 23 weeks. n = 6 mice per group at each time point. Student’s *t*-test was applied for statistical analysis. Area under the ITT curve shown right. **G** Serum insulin concentration of HFD-fed mice treated with honokiol or vehicle after 12 weeks of their respective diets. n = 6 mice per group. Student’s *t*-test was applied for statistical analysis. **H** GSVA pathway enrichment analysis related to cell death, inflammation, and fatty acid degradation differentially regulated by honokiol treatment. n = 5 mice per group. **I** Heatmap of gene expression profiles involved in cell damage and death, inflammation, and fatty acid degradation

We further analyzed the effect of honokiol on HFD-induced metabolic disorder. The white adipose tissue (WAT) and subcutaneous adipose tissue (SAT) weighed significantly less in honokiol-treated groups (Fig. 3A, B), with significantly decreased area and size of adipocytes and inflammatory cell infiltration in the WAT of honokiol-treated mice than controls on histopathological examination (Fig. 3C, D). Glucose tolerance testing (GTT) revealed a significant improvement after honokiol administration (Fig. 3E), as did insulin tolerance testing (ITT) (Fig. 3F). Furthermore, serum insulin concentrations also markedly decreased in honokiol-treated mice (Fig. 3G). Systematic transcriptome analysis of HFD mouse white adipose tissue showed significant therapeutic effects of honokiol with respect to cell damage, inflammation, and lipid metabolism pathways and associated gene expression (Fig. 3H, I). These results suggest that honokiol improves systemic metabolism.

### Honokiol blocks severe inflammation and fibrosis in mouse NASH models

**Fig. 4:**
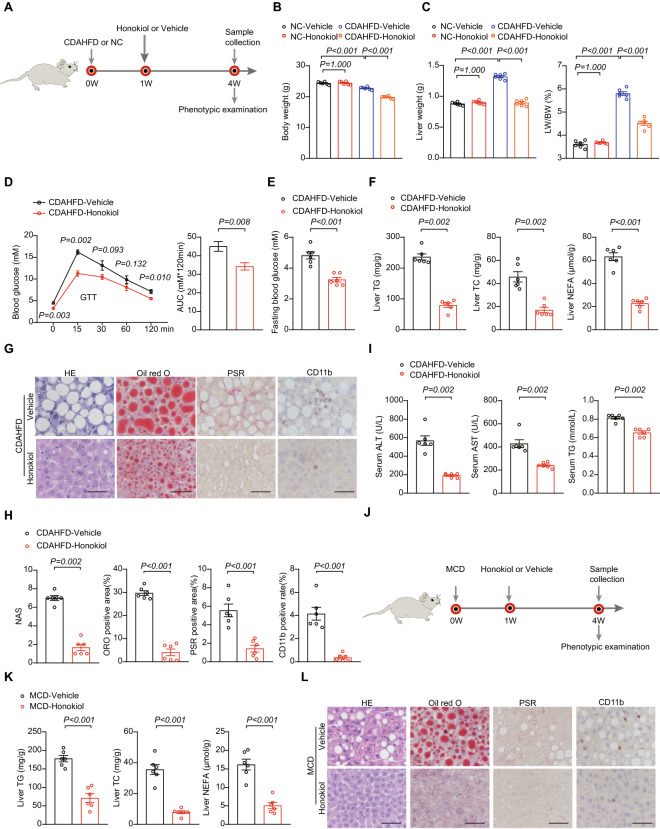

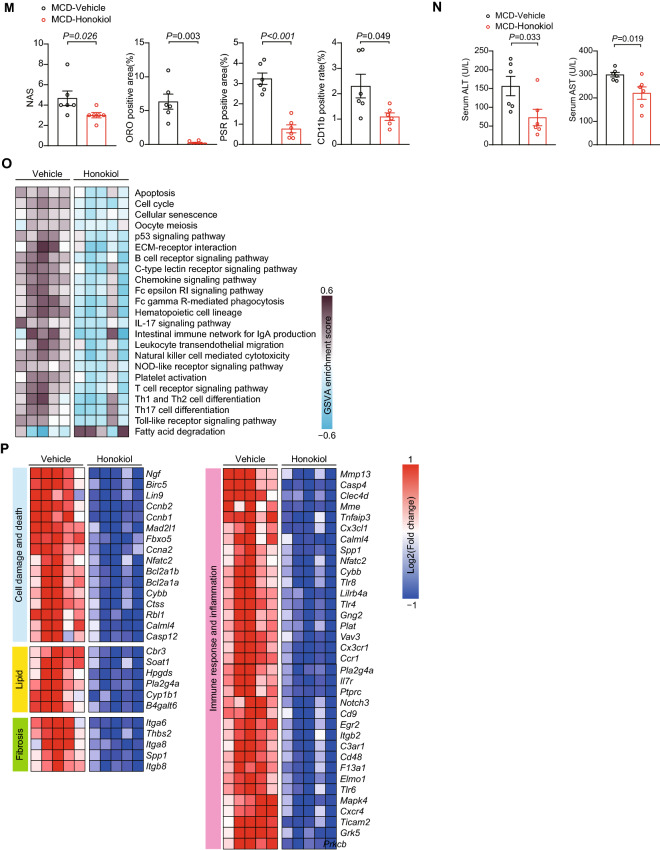
Honokiol protects against mouse NASH. **A** Schematic of the CDAHFD-induced NASH model and evaluating the therapeutic effects of honokiol in vivo (100 mg/kg). **B** Body weights of NC- or CDAHFD-fed mice treated with honokiol or vehicle three weeks after subjecting them to their respective diets for one week. n = 6 mice per group. One-way ANOVA was used for statistical analysis. **C** Liver weights and ratio of liver weight to body weight (LW/BW) of NC- or CDAHFD-fed mice treated with honokiol or CMC three weeks after subjecting them to their respective diets for one week. n = 6 mice per group. One-way ANOVA assay was used for statistical analysis. **D** Blood glucose concentrations during GTT and the AUC of GTT of CDAHFD-fed mice treated with vehicle or honokiol. **E** Fasting blood glucose (FBG) concentrations of CDAHFD-fed mice treated with vehicle or honokiol. n = 6 mice per group. Student’s *t*-test was applied for statistical analysis. **F** TG, TC and NEFA levels in the livers of CDAHFD-fed mice treated with honokiol or vehicle. n = 6 mice per group. Student’s *t*-test was applied for statistical analysis. **G** Representative images of indicated mouse liver sections stained with HE, ORO, PSR, and IHC for CD11b-positive cells. n = 6 mice per group. Scale bar 50 μm. **H** Results of NAS (HE) and quantitative analysis of ORO, PSR, and CD11b shown in (**G**). n = 6 mice per group. The Mann–Whitney U test was used for NAS and Student’s *t*-test was applied to ORO, PSR, and CD11b data. **I** Serum ALT and AST activity and serum TG concentrations in CDAHFD-fed mice treated with honokiol or vehicles. n = 6 mice per group. Student’s *t*-test was applied for statistical analysis. **J** Schematic of the MCD-induced NASH model and evaluating the therapeutic effects of honokiol in vivo (100 mg/kg). **K** TG, TC. and NEFA levels in the livers of MCD-fed mice treated with honokiol or vehicle. n = 6 mice per group. Student’s *t*-test was applied for statistical analysis. **L** Representative images of the indicated mouse liver sections stained with HE, ORO, PSR, and IHC for CD11b-positive cells. n = 6 mice per group. Scale bar 50 μm. **M** Results of NAS (HE) and quantitative analysis of ORO, PSR, and CD11b data shown in (**L)**. n = 6 mice per group. For statistical analysis, the Mann–Whitney U test was used for NAS and Student’s *t*-test was applied to ORO, PSR, and CD11b. **N** Serum ALT and AST activity of MCD-fed mice treated with honokiol or vehicle. n = 6 mice per group. Student’s *t*-test was applied for statistical analysis. **O** GSVA pathway enrichment analysis related to cell damage and death, inflammation, lipid metabolism, and fibrosis differentially regulated by honokiol treatment. n = 5 mice per group. **P** Heatmaps of gene expression profiles involved in cell damage and death, lipid metabolism, inflammation, and fibrosis

To further evaluate whether honokiol could block NASH progression, we applied a more severe NASH model induced by a choline-deficient, l-amino acid-defined high fat diet (CDAHFD). Compared with HFD models, CDAHFD-induced NASH models more closely resemble human NASH pathology in terms of ballooning and fibrosis, making them especially suitable for pharmacological interventions [29]. To initiate NASH progression, mice were subjected to CDAHFD for one week, followed by oral gavage with honokiol at 100 mg/kg every day along with CDAHFD feeding for another three weeks (Fig. 4A). Honokiol administration significantly decreased body weight, liver weight, and liver to body weight (LW/BW) ratio in CDAHFD-fed mice but had negligible impact in NC-fed mice (Fig. 4B, C). Both GTT and fasting blood glucose levels significantly improved on honokiol treatment (Fig. 4D, E). Moreover, hepatic lipid accumulation, fibrosis, and inflammatory cell infiltration were all significantly mitigated by honokiol administration (Fig. 4F–H). Consistent with the above effects, ALT, AST, and TG levels were all significantly lower in the honokiol-treated group (Fig. 4I).

To further clarify the beneficial effect of honokiol on averting NASH progression, we established the methionine and choline deficient diet (MCD)-induced NASH model. In this NASH model, mice were subjected to MCD for one week followed by oral gavage of honokiol at 100 mg/kg every day along with MCD feeding for another three weeks (Fig. 4J). In MCD-fed NASH mice, liver lipid accumulation, fibrosis, and inflammatory cell infiltrates were all significantly improved by honokiol administration (Fig. 4K–M). Serum ALT and AST were consistently and significantly lower in the honokiol-treated group (Fig. 4N). Global transcriptomic profiling of liver tissue revealed significant differences in cell damage, inflammation, lipid metabolism, and fibrosis pathways between vehicle and honokiol-treated mice fed by CDAHFD (Fig. 4O, P).

### Honokiol significantly activates AMPK in vitro and in vivo

**Fig. 5:**
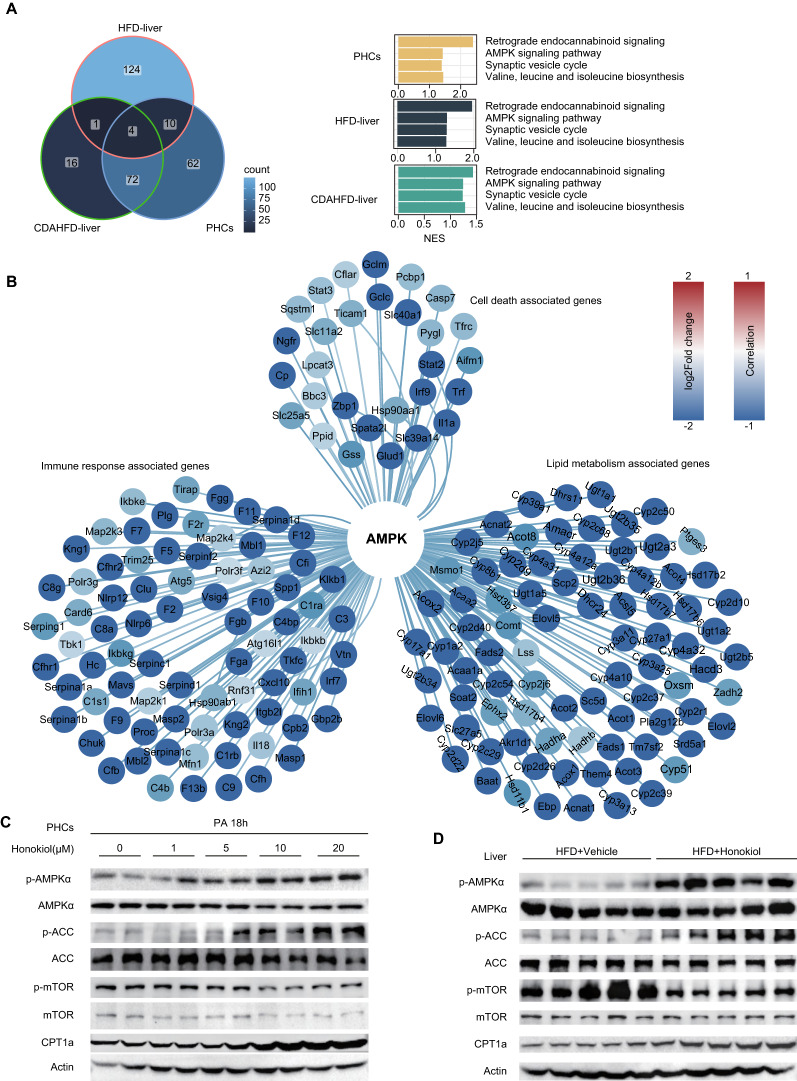

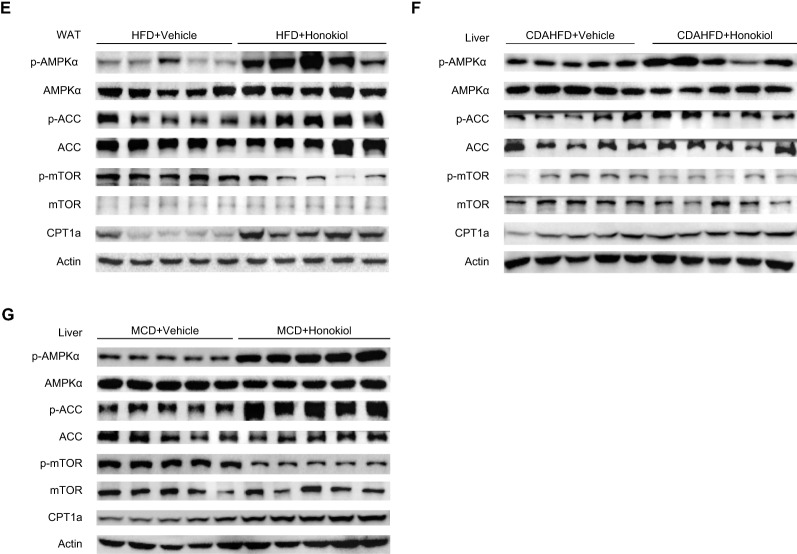
The AMPK signaling pathway is activated in vitro and in vivo. **A** The AMPK signaling pathway was one of the four common signaling pathway upregulated by honokiol in PHCs and in the livers of HFD- and CDAHFD-fed mice. **B** The AMPK signaling was negatively associated with cell death-associated genes, immune response-associated genes, and lipid metabolism-associated genes in HFD-fed mouse livers. **C** Western blots of p-AMPKα, AMPKα, p-ACC, ACC, p-mTOR, mTOR, and CPT1α in PHCs stimulated with palmitic acid (PA) for 18 h at the indicated concentrations. n = 3 replicates. **D** Western blots of p-AMPKα, AMPKα, p-ACC, ACC, p-mTOR, mTOR, and CPT1α in the livers of HFD-fed mice treated with vehicle or honokiol. n = 5 mice per group. **E** Western blots of p-AMPKα, AMPKα, p-ACC, ACC, p-mTOR, mTOR, and CPT1α in the WAT of HFD-fed mice treated with CMC or honokiol. n = 5 mice per group. **F** Western blots of p-AMPKα, AMPKα, p-ACC, ACC, p-mTOR, mTOR, and CPT1α in the livers of CDAHFD-fed mice treated with vehicle or honokiol. n = 5 mice per group. **G** Western blots of p-AMPKα, AMPKα, p-ACC, ACC, p-mTOR, mTOR, and CPT1α in the livers of MCD-fed mice treated with vehicle or honokiol. n = 5 mice per group

To explore the molecular mechanisms underlying the observed beneficial effects of honokiol, we interrogated the transcriptomic data obtained from our in vitro and in vivo models. Combined transcriptomic analysis suggested that activated AMPK signaling was one of the common pathways upregulated by honokiol (Fig. 5A). Moreover, correlation analysis also indicated that AMPK signaling was negatively related to cell death, inflammatory responses, and lipid metabolism in fatty liver settings (Fig. 5B). In line with the transcriptomics assay, Western blotting revealed that honokiol treatment activated AMPK in vitro (Fig. 5C). Furthermore, honokiol consistently activated AMPK and inhibited mTOR in both liver and WAT in the indicated mouse models (Fig. 5D–G). These results robustly demonstrate AMPK activation by honokiol and collectively point to AMPK activation as the molecular mechanism mediating its anti-NASH protective effects.

### AMPK activation is essential for honokiol-mediated liver protection

**Fig. 6:**
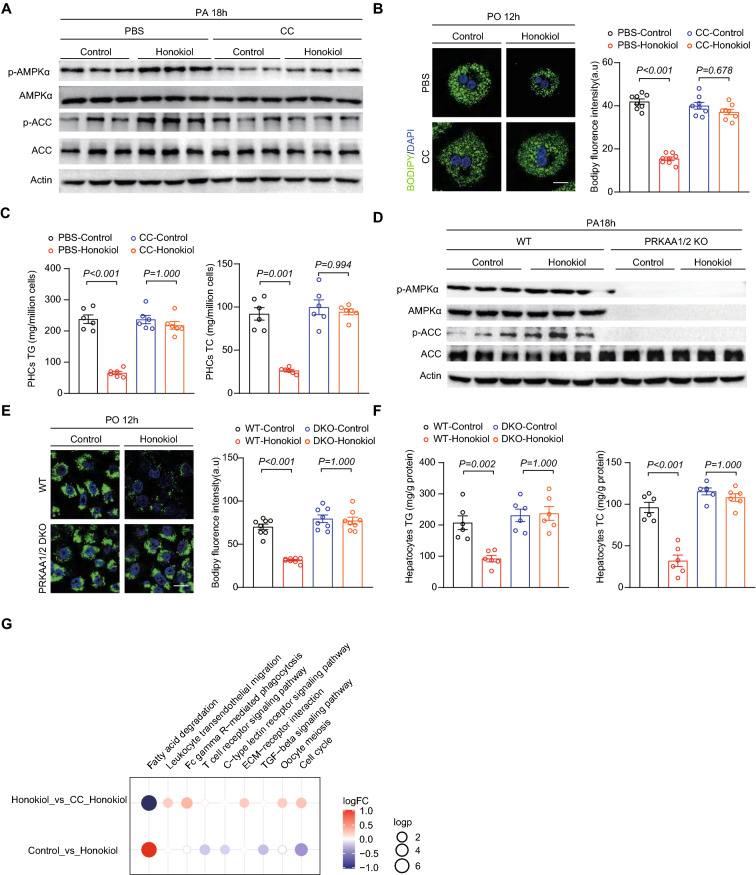
AMPK activation is required for honokiol-mediated beneficial effects in vitro. **A** Western blots of p-AMPKα, AMPKα, p-ACC, and ACC in primary hepatocytes challenged with PA for 18 h under the indicated conditions. n = 3 replicates **B** Representative images (left) of BODIPY staining and quantification (right) of lipid droplets in primary hepatocytes challenged with PO under the indicated conditions. n = 3 replicates. One-way ANOVA was used for statistical analysis. Scale bar 10 μm. **C** TG and TC of primary hepatocytes challenged with PO stimulation for 18 h under the indicated conditions. n = 6 mice per group. One-way ANOVA was used for statistical analysis. **D** Western blots of p-AMPKα, AMPKα, p-ACC, and ACC in WT or *PRKAA1/2* (encoding AMPKα1/2) double-knockout (DKO) hepatocytes challenged with PA for 18 h under the indicated conditions. **E** Representative images (left) of BODIPY staining and quantification (right) of lipid droplets in WT or *PRKAA1/2* DKO L02 hepatocytes challenged with PO for 12 h under the indicated conditions. n = 3 replicates. One-way ANOVA was used for statistical analysis. Scale bar 10 μm. **F** TG and TC of WT or *PRKAA1/2* DKO hepatocytes challenged with PO stimulation for 18 h under the indicated conditions. **G** Dot plot representing pairwise GSVA comparisons of transcriptomic data from primary hepatocytes challenged with PA for 18 h under the indicated conditions

To further demonstrate whether AMPKα activation is essential for the protective effects of honokiol, we cotreated primary hepatocytes with the AMPK activation inhibitor compound C (CC) and honokiol. CC significantly reduced the induction of AMPK activity and largely diminished the lipid-lowering effect by honokiol in primary hepatocytes (Fig. 6A–C). To further confirm the requirement of AMPK activation genetically, we generated *PRKAA1* and *PRKAA2* (expressing AMPKα1 and AMPKα2) double knockout (DKO) hepatocytes (Fig. 6D). Of note, AMPKα-DKO completely abolished the lipid-lowering effect of honokiol in hepatocytes (Fig. 6E, F). Furthermore, transcriptomic profiling firmly validated the reversal of pathways nvolved in lipid metabolism and inflammation by CC treatment (Fig. 6G). The collective pharmacological and genetic evidence suggest that AMPK activation is a necessary component for honokiol to exert a protective effect in vitro.

**Fig. 7:**
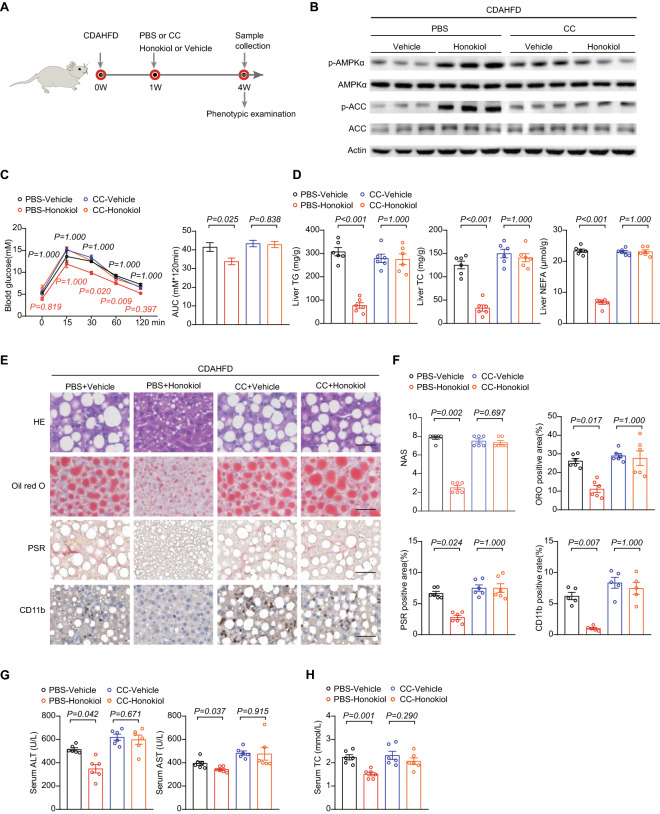

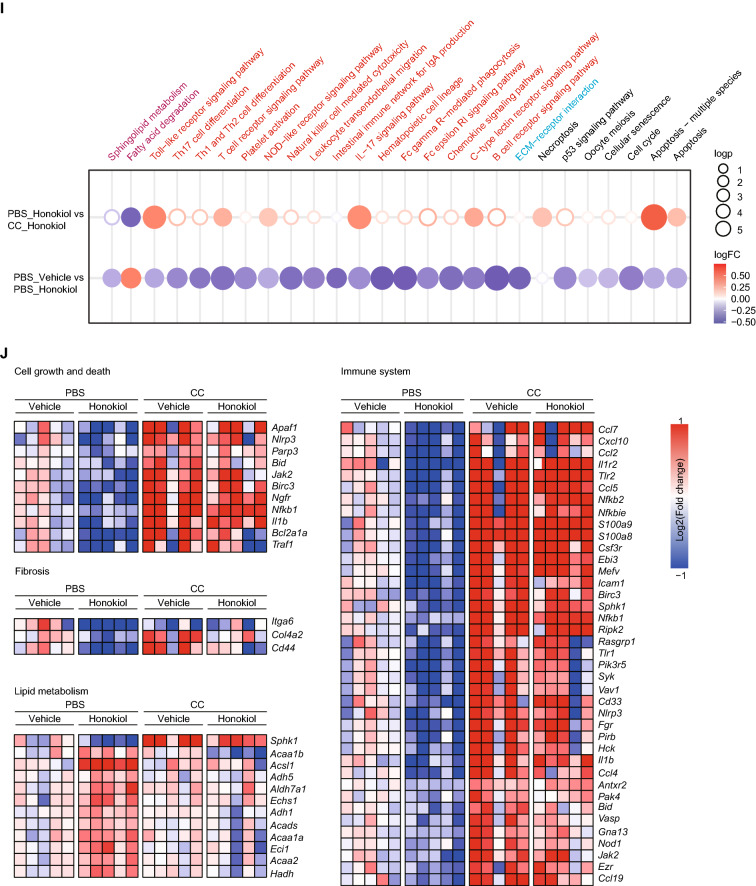
AMPK activation is required for honokiol-mediated beneficial effects in vivo. **A** Schematic of the experimental procedure used with mice fed a CDAHFD diet and treated with vehicle or honokiol (100 mg/kg) in the absence or presence of compound C (CC, 10 mg/kg, every other day, i.p.). **B** Western blots of p-AMPKα, AMPKα, p-ACC, and ACC of mice in the indicated groups. n = 3 mice per group. **C** The blood glucose concentration during GTT of CDAHFD-fed mice in the indicated groups. n = 6 mice per group. One-way ANOVA was applied for statistical analysis. *P*-values of the red color represent the comparison of PBS-vehicle vs PBS-honokiol, while black represents CC-vehicle vs CC-honokiol. **D** Liver contents of TG, TC, and NEFA of CDAHFD-fed mice in the indicated groups. n = 6 mice per group. The Kruskal–Wallis test was applied for statistical analysis. **E** Representative images of the indicated mouse liver sections stained with HE, ORO, PSR, and IHC for CD11b-positive cells. n = 6 mice per group. Scale bar, 50 μm. **F** Results of NAS (HE) and quantitative analysis of ORO, PSR, and CD11b data shown in (**E**). n = 6 mice per group. For statistical analysis, the Kruskal–Wallis test was used for NAS and one-way ANOVA was applied to ORO, PSR, and CD11b data. **G** Serum ALT and AST activity of CDAHFD-fed mice shown in the indicated groups. n = 6 mice per group. Student’s *t*-test was applied for statistical analysis. **H** Serum TC concentrations in CDAHFD-fed mice treated with the indicated groups. n = 6 mice per group. Student’s *t*-test was applied for statistical analysis. **I** Dot plot representing pairwise GSVA comparisons of transcriptomic data from CDAHFD-fed mice shown in the indicated groups. **J** Heatmap of transcriptomic data from CDAHFD-fed mice shown in the indicated groups

We next explored whether AMPK activation is vital to honokiol-mediated protective effects in vivo. We subjected CDAHFD-induced NASH mice to combined administration of CC (10 mg/kg/i.p. every other day) and honokiol (100 mg/kg every day, intragastric gavage) (Fig. 7A). CC effectively diminished honokiol-induced AMPK activation in the liver (Fig. 7B). CC treatment also abrogated the observed improvements in glucose intolerance by honokiol (Fig. 7C). The beneficial effects of honokiol on liver lipid accumulation, fibrosis, and inflammatory cell infiltration were also significantly reversed (Fig. 7D–F). CC treatment also reduced honokiol-induced improvements in serum markers of liver function and lipid metabolism (Fig. 7G, H). Finally, transcriptomic data systematically showed CC-induced reversal of the pathways and gene expression profiles involved in lipid metabolism, inflammation, fibrosis, and cell damage (Fig. 7I, J). All the evidence led to the conclusion that AMPK activation is critical component of honokiol-mediated hepatic protection.

### Honokiol activates AMPK by directly binding to AMPKγ1 and acts as an AMPK complex agonist

**Fig. 8:**
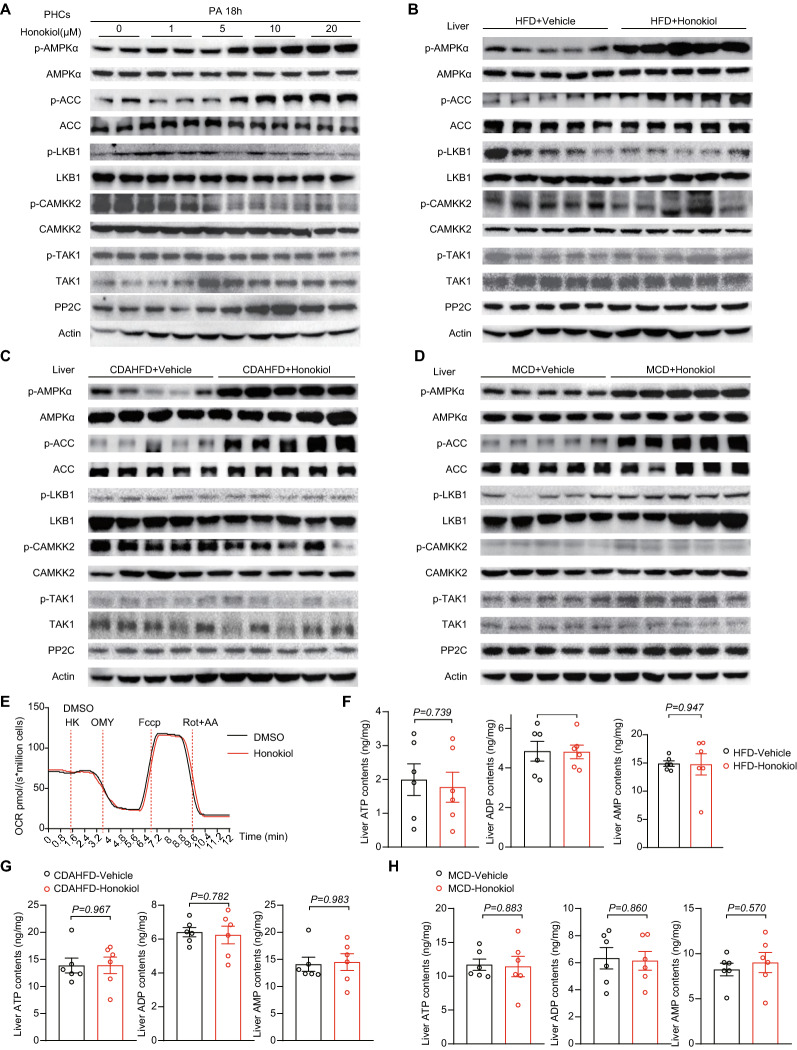
AMPK activation is independent of classical pathways. **A** Western blots of p-AMPKα, AMPKα, p-ACC, ACC, p-LKB1, LKB1, p-CAMKK2, CAMKK2, p-TAK1, TAK1, and PP2C of primary hepatocytes subjected to PA stimulation at the indicated conditions. n = 3 replicates. **B** Western blots of p-AMPKα, AMPKα, p-ACC, ACC, p-LKB1, LKB1, p-CAMKK2, CAMKK2, p-TAK1, TAK1, and PP2C in the livers of HFD-fed mice treated with vehicle or honokiol. n = 5 mice per group. **C** Western blots of p-AMPKα, AMPKα, p-ACC, ACC, p-LKB1, LKB1, p-CAMKK2, CAMKK2, p-TAK1, TAK1, and PP2C in the livers of CDAHFD-fed mice treated with vehicle or honokiol. n = 5 mice per group. **D** Western blots of p-AMPKα, AMPKα, p-ACC, ACC, p-LKB1, LKB1, p-CAMKK2, CAMKK2, p-TAK1, TAK1, and PP2C in the livers of MCD-fed mice treated with vehicle or honokiol. n = 5 mice per group. **E** Representative image of the oxygen consumption rate (OCR) of primary hepatocytes subjected to DMSO or honokiol at the indicated conditions. n = 3 replicates. Omy, oligomycin, F1F0 ATP synthase inhibitor. FCCP, mitochondrial uncoupler. Rot, retenone, complex I inhibitor. AA, antimycin A, complex II inhibitor. **F** ATP, ADP, and AMP in the livers of HFD-fed mice treated with vehicle or honokiol. n = 6 mice per group. Student’s *t*-test was applied for statistical analysis. **G** ATP, ADP, and AMP in the livers of CDAHFD-fed mice treated with vehicle or honokiol. n = 6 mice per group. Student’s *t*-test was applied for statistical analysis. **H** ATP, ADP, and AMP in the livers of MCD-fed mice treated with vehicle or honokiol. n = 6 mice per group. Student’s *t*-test was applied for statistical analysis

To further explore the exact molecular mechanisms underpinning honokiol’s activation of AMPK signaling, we examined the impact of honokiol on well-established upstream regulators of AMPK. However, honokiol showed negligible influence on the expression and phosphorylation of liver kinase B1 (LKB1), transforming growth factor beta-activated kinase 1 (TAK1), calcium/calmodulin-dependent protein kinase kinase 2 (CAMKK2), and no decrease in the negative regulator protein phosphatases 2C (PP2C) in hepatocytes or fatty livers compared with blank controls (Fig. 8A–D). As the AMPK complex directly senses cellular energy status, we hypothesized that these negative findings might be related to the direct influence of honokiol on cellular respiration and subsequent changes in cellular ATP, ADP, and AMP. However, the oxygen consumption rate (OCR) of hepatocytes was not significantly altered upon treatment with honokiol (Fig. 8E). Similarly, liver ATP, ADP, and AMP levels were not significantly altered by honokiol in fatty livers in diet-induced mouse models (Fig. 8F–H). This evidence collectively suggests the possibility of a previously unknown mechanism through which honokiol activates AMPK.

**Fig. 9:**
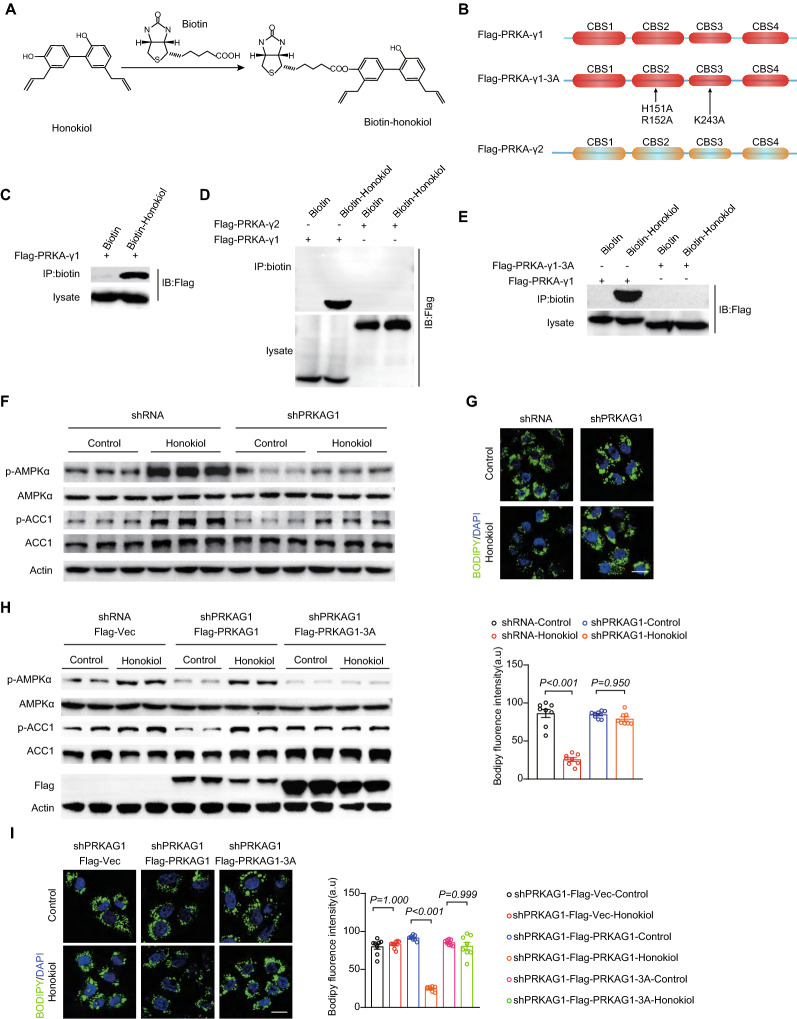

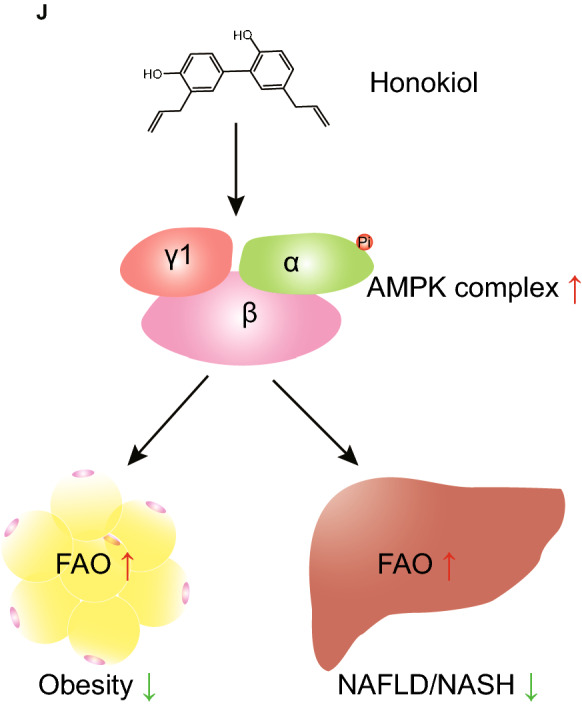
AMPK activation is independent of classical pathways. **A** The structure of honokiol and the synthesis of biotin-linked honokiol. **B** Schematic showing the construction of plasmids expressing 3X-FLAG-*PRKAG1*, 3X-FLAG-*PRKAG2*, and 3X-FLAG-*PRKAG1*-3A, respectively. **C** Representative gels showing the binding of honokiol to Flag-AMPKγ1 in 293 T cells. n = 3 replicates. **D** Representative gels showing the binding of honokiol to Flag-labeled AMPKγ1 and Flag-labeled AMPKγ2. n = 3 replicates. **E** Representative gels showing the binding of honokiol to Flag-labeled AMPKγ1 and Flag-labeled AMPKγ1-3A. n = 3 replicates. **F** Western blots of p-AMPKα, AMPKα, p-ACC, and ACC of shRNA hepatocytes or sh*PRKAG1* hepatocytes at the indicated conditions. n = 3 replicates. **G** Representative images (left) of BODIPY staining and quantification (right) of lipid droplets in shRNA hepatocytes or sh*PRKAG1* hepatocytes challenged with PO for 12 h under the indicated conditions. n = 3 replicates. One-way ANOVA was used for statistical analysis. Scale bar 10 μm. **H** Western blots of p-AMPKα, AMPKα, p-ACC, and ACC of shRNA hepatocytes transfected with empty vector and sh*PRKAG1* hepatocytes transfected with Flag-*PRKAG1* or Flag-*PRKAG1*-3A plasmids and challenged with PA for 18 h under the indicated conditions. n = 3 replicates. **I** Representative images (left) of BODIPY staining and quantification (right) of lipid droplets in sh*PRKAG1* L02 hepatocytes transfected with empty vector, Flag-*PRKAG1*, or Flag-*PRKAG1*-3A plasmids and challenged with PO for 12 h under the indicated conditions. n = 3 replicates. Scale bar 10 μm. **J** Schematic showing the mechanism of honokiol-mediated activation of the AMPK complex and beneficial effects of honokiol in ameliorating obesity and NASH progression

Docking analysis suggested that honokiol might directly bind to the AMPKγ1 subunit [30], potentially explaining how honokiol activates AMPK. To test this prediction, we chemically linked biotin to honokiol and constructed plasmids expressing AMPKγ1 (Fig. 9A). Since AMPKγ2 is predominantly expressed in the liver, we also created an AMPKγ2 plasmid (Fig. 9B). Streptavidin–biotin binding assays showed that honokiol significantly interacted with AMPKγ1 but not with AMPKγ2 (Fig. 9C, D). Honokiol binds AMPKγ1 at histidine (151), arginine (152), and lysine (243), since mutation of these 3 amino acid sites largely abolished the interaction between honokiol and AMPKγ1 (Fig. 9E). Importantly, AMPKγ1 knockdown significantly reduced AMPK activation by honokiol, and consequently the lipid-lowering effects of honokiol (Fig. 9F, G). This suggests that AMPKγ1 is necessary for the full activation of AMPK by honokiol. Moreover, in sh*PRKAG1* cells, rescue of honokiol-induced AMPK activation and its subsequent protective effect only occurred in cells supplemented with WT-AMPKγ1, instead of the AMPKγ1-3A mutant (Fig. 9H, I). Our research suggests that honokiol could potentially be an AMPK complex agonist through direct binding to AMPKγ1 (Fig. 9J).

## Discussion

NAFLD and related metabolic syndrome have become major disease burdens worldwide, and approved pharmacological interventions for these conditions are lacking. The failure of promising drug candidates in phase II or phase III clinical trials over the last three years further emphasizes the urgent need for new, effective, and safe agents for NASH therapy. FDA-approved drug libraries provide a highly efficient strategy for anti-NASH drug screening and development, benefiting from the well-tested safety and pharmacology of the included compounds. Here we identified honokiol as an AMPK agonist that ameliorated NASH and metabolic syndrome. Intriguingly, honokiol did not inhibit lipid accumulation, inflammation, and cell damage via classical upstream AMPK activation pathways, but instead through direct interaction with AMPKγ1 and subsequent phosphorylation.

Honokiol is a pleiotropic compound found in magnolia plants, and it is used in traditional Chinese medicine for the treatment of several diseases. Previous studies have reported that honokiol is useful for the treatment of tumors, sepsis-associated acute lung injury, neurodegenerative diseases, and cardiomyopathy [31]. The main beneficial effects of honokiol are related to its ability to induce apoptosis, reduce inflammation, and scavenge harmful oxidizing agents. Moreover, previous studies have also reported that honokiol can ameliorate hepatocyte lipotoxicity and macrophage polarization in the liver [21–24]. It also has a reported anti-obesity effect [32, 33]. However, previous studies have not fully established the detailed molecular events and gene expression profiles related to the phenotypes induced by honokiol treatment. Based on the results of our present study, honokiol represents a promising drug candidate for metabolic disorder-related diseases via a previously unappreciated molecular mechanism. Notably, there might be other mechanisms, except from AMPK signaling, also involved in the beneficial effects of honokiol against NAFLD progression. In our experiments, both AMPK signaling and Retrograde endocannabinoid signaling are enriched, the latter having been associated with neuron diseases [34] but its implication in NAFLD yet to be investigated. It is essential to conduct further research to determine if honokiol can ameliorate NAFLD and metabolic syndrome through other pathways, such as retrograde endocannabinoid signaling.

Hepatic and adipose tissue lipid accumulation results from an imbalance between lipid production and utilization [35]. As noted above, our multi-transcriptomic analysis further showed that honokiol protected against obesity/NAFLD/NASH by promoting fatty acid oxidation (FAO), a key metabolic pathway for fatty acids. Further analysis of signaling pathways regulating FAO revealed AMPK activation. AMPK is a master regulator of nutrient metabolism, including lipid synthesis and degradation [36]. AMPK activation has been shown to protect against NASH [37–42], obesity [43], and type 2 diabetes [44, 45]. Notably, AMPK activation in the intestine by nicotine could aggravate NASH by increasing intestinal ceramide formation [46]. We confirmed activation of AMPK and its targets by honokiol [21, 24, 33], consistent with its protective effects against NAFLD and NASH in vitro and in vivo. Through both genetic and pharmacological methods, we demonstrated that the protective effect of honokiol in NAFLD/NASH depends on AMPK activation. Transcriptomic analysis suggested that compound C (CC) treatment significantly reversed the gene expression profile regulated by honokiol administration.

As reported previously, AMPK activation is tightly regulated by upstream kinases and phosphatases [8]. However, we found that honokiol did not rely on its classical regulators for activation of AMPK. Molecular docking analysis and biotin-avidin affinity capture of honokiol and AMPK complex indicated that honokiol could directly bind to the AMPKγ1 subunit, thus activating the AMPK complex. After point mutation of predicted binding sites, enrichment of the AMPKγ1-3A mutant by honokiol largely decreased. These experimental results further suggest honokiol can bind to AMPKγ1 to activate the AMPK complex. In *PRKAG1* (gene expressing AMPKγ1)-knockdown hepatocytes, we found a marked reduction in honokiol-induced AMPK activation, an effect that could be rescued by supplementation with AMPKγ1 but not with the AMPKγ1-3A mutant. These findings suggest direct targeting AMPKγ1 by honokiol to activate the AMPK complex. Consistent with this, liver-specific gain-of-function mutations in AMPKγ1 or direct targeting of AMPKγ1 with small molecules have shown protective effects against NASH [12, 47, 48] and liver glucose production [49], highlighting the importance of AMPKγ1 in the subsequent protective effects of AMPK activation. These results suggest that honokiol appears to act as an AMPK complex agonist that can be applied to several AMPK inactivation-mediated pathologies.

Hepatic and adipose tissue lipid accumulation occurs through an imbalance between lipid production and utilization [35]. Our multi-transcriptomic analysis showed that honokiol benefited obesity/NAFLD/NASH by promoting FAO, which is a key metabolic pathway for fatty acids. Further analysis of signaling pathways regulating FAO revealed AMPK activation. AMPK is a master regulator of nutrient metabolism, including lipid synthesis and degradation [36]. Inactivation of AMPK is a major hallmark of metabolic diseases [25, 41, 50–53], while activating AMPK has been shown to protect against NASH [37–42], obesity [43] and type 2 diabetes [44, 45]. However, excessive activation of AMPK might lead to unwanted side-effects, for instance AMPK activation in the intestine by nicotine from tobacco could aggravate NASH by increasing intestinal ceramide formation [46]. Furthermore, long-term administration of pan-AMPK agonists is causally related to cardiac hypertrophy [54]. In the present study, using genetic and pharmacological methods, we demonstrated that the protective effect of honokiol on NASH and its related metabolic diseases is dependent on AMPK activation. More importantly, we did not observe any side effects of honokiol on the cardiovascular system, which might be due to the specific regulation of AMPK by honokiol.

## Conclusions

In summary, here we introduce a treatment that may be suitable for the entire spectrum of NASH and metabolic syndrome. Honokiol effectively prevented lipid accumulation, cell damage, and immune responses both in vitro and in vivo*.* In-depth analysis of the molecular mechanisms regulating FAO uncovered significant activation of AMPK, which was required for honokiol’s mechanism of action in pharmacological and genetic studies. Of note, honokiol-mediated activation of the AMPK complex did not rely on its classical regulators, instead acting as an AMPK complex agonist via directly binding to AMPKγ1 subunit. Thus, our findings add new insight that targeting AMPKγ1 with small molecular agents could be a potential treatment for obesity, NAFLD, and NASH without adverse effects.

## Supplementary Information

**Additional file 1: Fig. S1.** Lipid metabolism pathway influenced by honokiol treatment. (A) Heatmaps of gene expression associated with multiple lipid metabolism pathways of liver from HFD-fed mice.

## Data Availability

The datasets used and/or analyzed during the current study are available from the corresponding author on reasonable request.

## Abbreviations

AMPK: AMP activated protein kinase
CC: Compound C
CDAHFD: Choline-deficient, L-amino acid-defined high-fat diet
FAO: Fatty acid oxidation
FBC: Fasting blood glucose
GTT: Glucose tolerance test
HFD: High fat diet
ITT: Insulin tolerance test
LW/BW: Liver weight to body weight ratio
MCD: Methionine choline deficient diet
NAFLD: Non-alcoholic fatty liver disease
NAS: NAFLD activity scoring
NASH: Non-alcoholic steatohepatitis
NEFA: Non-esterified fatty acids
OCR: Oxygen consumption rate
PA: Palmitic acid
PHCs: Primary hepatocytes
SAT: Subcutaneous adipose tissue
WAT: White adipose tissue

## References

[1] Powell EE, Wong VW, Rinella M (2021). Non-alcoholic fatty liver disease. Lancet.

[2] Paik JM (2020). Changes in the global burden of chronic liver diseases from 2012 to 2017: the growing impact of NAFLD. Hepatology.

[3] Zhou F (2019). Unexpected rapid increase in the Burden of NAFLD in China From 2008 to 2018: a systematic review and meta-analysis. Hepatology.

[4] Estes C (2018). Modeling NAFLD disease burden in China, France, Germany, Italy, Japan, Spain, United Kingdom, and United States for the period 2016–2030. J Hepatol.

[5] Estes C (2018). Modeling the epidemic of nonalcoholic fatty liver disease demonstrates an exponential increase in burden of disease. Hepatology.

[6] Cai J (2020). Nonalcoholic fatty liver disease pandemic fuels the upsurge in cardiovascular diseases. Circ Res.

[7] Chen Z (2021). Nonalcoholic fatty liver disease: an emerging driver of cardiac arrhythmia. Circ Res.

[8] Steinberg GR, Hardie DG (2022). New insights into activation and function of the AMPK. Nat Rev Mol Cell Biol.

[9] Trefts E, Shaw RJ (2021). AMPK: restoring metabolic homeostasis over space and time. Mol Cell.

[10] Steinberg GR, Carling D (2019). AMP-activated protein kinase: the current landscape for drug development. Nat Rev Drug Discov.

[11] Roustan V (2016). An evolutionary perspective of AMPK–TOR signaling in the three domains of life. J Exp Bot.

[12] Woods A (2017). Liver-specific activation of ampk prevents steatosis on a high-fructose diet. Cell Rep.

[13] Selleckchem.com. *FDA-approved Drug Library*.

[14] Lee YJ (2011). Therapeutic applications of compounds in the Magnolia family. Pharmacol Ther.

[15] Cho JH (2015). Multifunctional effects of honokiol as an anti-inflammatory and anti-cancer drug in human oral squamous cancer cells and xenograft. Biomaterials.

[16] Xiang H (2017). Chinese herbal medicines attenuate acute pancreatitis: pharmacological activities and mechanisms. Front Pharmacol.

[17] Khatoon F (2022). Pharmacological features, health benefits and clinical implications of honokiol. J Biomol Struct Dyn.

[18] Shen JL (2010). Honokiol and magnolol as multifunctional antioxidative molecules for dermatologic disorders. Molecules.

[19] Feng RB (2018). Gallic acid, a natural polyphenol, protects against tert-butyl hydroperoxide- induced hepatotoxicity by activating ERK-Nrf2-Keap1-mediated antioxidative response. Food Chem Toxicol.

[20] Sarrica A (2018). Safety and toxicology of magnolol and honokiol. Planta Med.

[21] Seo MS (2015). Honokiol activates the LKB1-AMPK signaling pathway and attenuates the lipid accumulation in hepatocytes. Toxicol Appl Pharmacol.

[22] Zhai T (2020). Honokiol alleviates methionine-choline deficient diet-induced hepatic steatosis and oxidative stress in C57BL/6 Mice by regulating CFLAR-JNK pathway. Oxid Med Cell Longev.

[23] Zhong X, Liu H (2018). Honokiol attenuates diet-induced non-alcoholic steatohepatitis by regulating macrophage polarization through activating peroxisome proliferator-activated receptor gamma. J Gastroenterol Hepatol.

[24] Liu J (2021). Honokiol attenuates lipotoxicity in hepatocytes via activating SIRT3-AMPK mediated lipophagy. Chin Med.

[25] Jian C (2020). Low-dose sorafenib acts as a mitochondrial uncoupler and ameliorates nonalcoholic steatohepatitis. Cell Metab.

[26] Zhang CS (2017). Fructose-1,6-bisphosphate and aldolase mediate glucose sensing by AMPK. Nature.

[27] Zhang XJ (2021). A small molecule targeting ALOX12-ACC1 ameliorates nonalcoholic steatohepatitis in mice and macaques. Sci Transl Med.

[28] Kleiner DE (2005). Design and validation of a histological scoring system for nonalcoholic fatty liver disease. Hepatology.

[29] Farrell G (2019). Mouse models of nonalcoholic steatohepatitis: toward optimization of their relevance to human nonalcoholic steatohepatitis. Hepatology.

[30] Liu, H, et al., The glucotoxicity protecting effect of honokiol in human hepatocytes via directly activating AMPK. Frontiers in Nutrition. 9:1043009. 10.3389/fnut.2022.1043009. 10.3389/fnut.2022.1043009 PMC9716082 36466390

[31] Rauf A (2021). Honokiol: a review of its pharmacological potential and therapeutic insights. Phytomedicine.

[32] Ding Y (2019). Honokiol ameliorates high-fat-diet-induced obesity of different sexes of mice by modulating the composition of the gut microbiota. Front Immunol.

[33] Ding Y (2021). Honokiol alleviates high-fat diet-induced obesity of mice by inhibiting adipogenesis and promoting white adipose tissue browning. Animals.

[34] Lu HC, Mackie K (2016). An introduction to the endogenous cannabinoid system. Biol Psychiatry.

[35] Loomba R, Friedman SL, Shulman GI (2021). Mechanisms and disease consequences of nonalcoholic fatty liver disease. Cell.

[36] Herzig S, Shaw RJ (2018). AMPK: guardian of metabolism and mitochondrial homeostasis. Nat Rev Mol Cell Biol.

[37] Jian C (2020). Low-dose sorafenib acts as a mitochondrial uncoupler and ameliorates nonalcoholic steatohepatitis. Cell Metab.

[38] Zhao P (2020). An AMPK-caspase-6 axis controls liver damage in nonalcoholic steatohepatitis. Science.

[39] Hu M (2021). Salidroside activates the AMP-activated protein kinase pathway to suppress nonalcoholic steatohepatitis in mice. Hepatology.

[40] Lan T (2021). Cordycepin ameliorates nonalcoholic steatohepatitis by activation of the Amp-activated protein kinase signaling pathway. Hepatology.

[41] Lin Q (2021). Activating adenosine monophosphate-activated protein kinase mediates fibroblast growth factor 1 protection from nonalcoholic fatty liver disease in mice. Hepatology.

[42] Wan J (2021). Gastrodin improves nonalcoholic fatty liver disease through activation of the adenosine monophosphate-activated protein kinase signaling pathway. Hepatology.

[43] Mottillo EP (2016). Lack of adipocyte AMPK exacerbates insulin resistance and hepatic steatosis through brown and beige adipose tissue function. Cell Metab.

[44] Deng X (2015). Docosahexaenoic acid inhibits proteolytic processing of sterol regulatory element-binding protein-1c (SREBP-1c) via activation of AMP-activated kinase. Biochim Biophys Acta.

[45] Aguilar-Recarte D (2022). Knocking on GDF15's door for the treatment of type 2 diabetes mellitus. Trends Endocrinol Metab.

[46] Chen B (2022). Gut bacteria alleviate smoking-related NASH by degrading gut nicotine. Nature.

[47] Yang Y (2021). Naringenin attenuates non-alcoholic fatty liver disease by enhancing energy expenditure and regulating autophagy via AMPK. Front Pharmacol.

[48] Wu C (2014). Cordycepin activates AMP-activated protein kinase (AMPK) via interaction with the γ1 subunit. J Cell Mol Med.

[49] An H (2020). The importance of the AMPK gamma 1 subunit in metformin suppression of liver glucose production. Sci Rep.

[50] Zhu X (2019). Berberine attenuates nonalcoholic hepatic steatosis through the AMPK-SREBP-1c-SCD1 pathway. Free Radic Biol Med.

[51] Rao Y (2019). Bouchardatine analogue alleviates non-alcoholic hepatic fatty liver disease/non-alcoholic steatohepatitis in high-fat fed mice by inhibiting ATP synthase activity. Br J Pharmacol.

[52] Song L (2022). FGF4 protects the liver from nonalcoholic fatty liver disease by activating the AMP-activated protein kinase-Caspase 6 signal axis. Hepatology.

[53] Castaño D (2014). Cardiotrophin-1 eliminates hepatic steatosis in obese mice by mechanisms involving AMPK activation. J Hepatol.

[54] Myers RW (2017). Systemic pan-AMPK activator MK-8722 improves glucose homeostasis but induces cardiac hypertrophy. Science.

