# Supplementary material for: Honokiol acts as an AMPK complex agonist therapeutic in non-alcoholic fatty liver disease and metabolic syndrome
Source: Chin Med. 2023 Mar 17;18:30. doi: 10.1186/s13020-023-00729-5 (PMC10024454; doi:10.1186/s13020-023-00729-5)
Supplement: Supplementary file 1 — Additional file 1: Fig. S1. Lipid metabolism pathway influenced by honokiol treatment. (A) Heatmaps of gene expression associated with multiple lipid metabolism pathways of liver from HFD-fed mice. [file 13020_2023_729_MOESM1_ESM.docx]

##
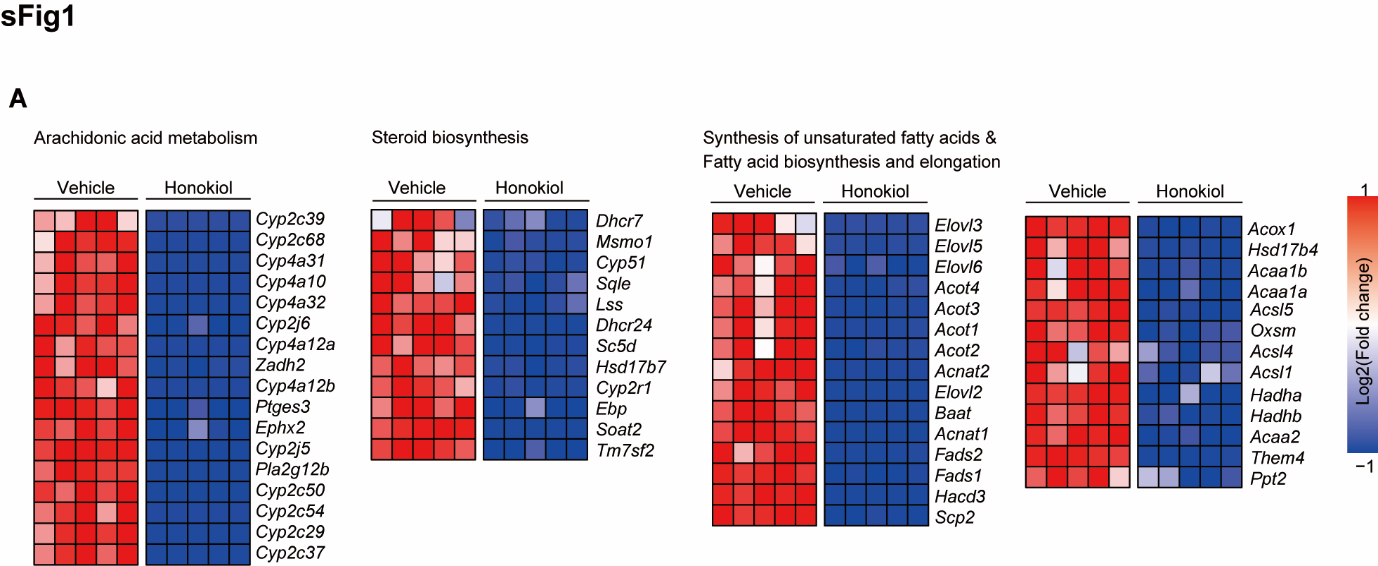


## Figure legends

**Fig. S1. Lipid metabolism pathway influenced by honokiol treatment. (A)** Heatmaps of gene expression associated with multiple lipid metabolism pathways of liver from HFD-fed mice.
